# Embodied Cognition is Not What you Think it is

**DOI:** 10.3389/fpsyg.2013.00058

**Published:** 2013-02-12

**Authors:** Andrew D. Wilson, Sabrina Golonka

**Affiliations:** ^1^School of Social, Psychological and Communication Sciences, Leeds Metropolitan UniversityLeeds, UK

**Keywords:** embodied cognition, dynamical systems, replacement hypothesis, robotics, outfielder problem, A-not-B error, language

## Abstract

The most exciting hypothesis in cognitive science right now is the theory that cognition is embodied. Like all good ideas in cognitive science, however, embodiment immediately came to mean six different things. The most common definitions involve the straight-forward claim that “states of the body modify states of the mind.” However, the implications of embodiment are actually much more radical than this. If cognition can span the brain, body, and the environment, then the “states of mind” of disembodied cognitive science won’t exist to be modified. Cognition will instead be an extended system assembled from a broad array of resources. Taking embodiment seriously therefore requires both new methods and theory. Here we outline four key steps that research programs should follow in order to fully engage with the implications of embodiment. The first step is to conduct a task analysis, which characterizes from a first person perspective the specific task that a perceiving-acting cognitive agent is faced with. The second step is to identify the task-relevant resources the agent has access to in order to solve the task. These resources can span brain, body, and environment. The third step is to identify how the agent can assemble these resources into a system capable of solving the problem at hand. The last step is to test the agent’s performance to confirm that agent is actually using the solution identified in step 3. We explore these steps in more detail with reference to two useful examples (the outfielder problem and the A-not-B error), and introduce how to apply this analysis to the thorny question of language use. Embodied cognition is more than we think it is, and we have the tools we need to realize its full potential.

## Introduction

The most exciting idea in cognitive science right now is the theory that cognition is *embodied*. It is, in fact one of the things interested lay people know about cognitive science, thanks to many recent high profile experiments. These experiments claim to show (1) how cognition can be influenced and biased by states of the body (e.g., Eerland et al., 2011) or the environment (Adam and Galinsky, 2012) or (2) that abstract cognitive states are grounded in states of the body and using the former affects the latter (e.g., Lakoff and Johnson, 1980, 1999; Miles et al., 2010).

The problem, however, is that *this is not really what embodied cognition is about*. Embodiment is the surprisingly radical hypothesis that the brain is not the sole cognitive resource we have available to us to solve problems. Our bodies and their perceptually guided motions through the world do much of the work required to achieve our goals, *replacing* the need for complex internal mental representations. This simple fact utterly changes our idea of what “cognition” involves, and thus embodiment is not simply another factor acting on an otherwise disembodied cognitive processes.

Many cognitive scientists, see this claim occupying the extreme end of an embodiment continuum, and are happy with the notion that there can be many co-existing notions of embodiment – maybe three (Shapiro, 2011) or even six (Wilson, 2002). Why rule out other research programs that seem to be showing results? Why not have one strand of embodied cognition research that focuses on how cognition can be biased by states of the body, and another strand that focuses on brain-body-environment cognitive systems? The issue is that the former type of research does not follow through on the necessary consequences of allowing cognition to involve more than the brain. These consequences, we will argue, lead *inevitably* to a radical shift in our understanding of what cognitive behavior is made from. This shift will take cognitive science away from tweaking underlying competences and toward understanding how our behavior emerges from the real time interplay of task-specific resources distributed across the brain, body, and environment, coupled together via our perceptual systems.

This paper will proceed as follows. After laying out the standard cognitive psychological approach to explaining behavior, we’ll briefly point to some interesting lines of empirical research from robotics and animal cognition that support the stronger *replacement hypothesis* of embodied cognition (Shapiro, 2011). We’ll then lay out a recommended research strategy based on this work. Specifically, we will detail how to use a *task analysis* to identify the cognitive requirements of a task and the resources (in brain, body, and environment) available to fill these requirements. According to this analysis, it is the job of an empirical research program to find out which of the available resources the organism is actually using, and how they have been assembled, coordinated, and controlled into a *smart*, *task-specific device* for solving the problem at hand (Runeson, 1977; Bingham, 1988). We’ll focus on two classic examples in detail: *the outfielder problem* (e.g., McBeath et al., 1995) and the *A-not-B task* (e.g., Thelen et al., 2001). We’ll then contrast this task-specific approach with some embodied cognition research in the standard cognitive psychology mold, and see how this latter research fails to successfully motivate any role for the body or environment, let alone the one identified in the research. Finally, we’ll conclude with some thoughts on how to begin to apply this approach to one of the harder problems in cognitive science, specifically language use. Language is the traditional bête noir of this more radical flavor of embodiment, and our goal in this final section will be to demonstrate that, with a little work, a truly embodied analysis of language can, in fact, get off the ground.

## Standard Cognitive Explanations for Behavior

The insight of early cognitive psychologists was that our behavior appears to be mediated by something internal to the organism. The classic example is Chomsky’s (1959) critique of “Verbal Behavior” (Skinner, 1957) in which he argues that language learning and use cannot be explained without invoking mental structures (in this case, innate linguistic capabilities). In general, the theoretical entities cognitive psychologists invoke to do this internal mediation are *mental representations*.

At the time these ideas were taking off, research on perception suggested that our perceptual access to the world wasn’t very good (see Marr, 1982; Rock, 1985 for reviews). This creates the following central problem for representations to solve. The brain is locked away inside our heads with only impoverished, probabilistic perceptual access to the world, but it has the responsibility of coordinating rapid, functional, and successful behavior in a dynamic physical and social environment. Because perception is assumed to be flawed, it is not considered a central resource for solving tasks. Because we only have access to the environment via perception, the environment also is not considered a central resource. This places the burden entirely on the brain to act as a storehouse for skills and information that can be rapidly accessed, parameterized, and implemented on the basis of the brain’s best guess as to what is required, a guess that is made using some optimized combination of sensory input and internally represented knowledge. This job description makes the content of internal cognitive representations the most important determinant of the structure of our behavior. Cognitive science is, therefore, in the business of *identifying this content* and *how it is accessed and used* (see Dietrich and Markman, 2003 for a discussion of this).

Advances in perception-action research, particularly Gibson’s work on direct perception (Gibson, 1966, 1979), changes the nature of the problem facing the organism. Perception is not critically flawed. In fact, we have extremely high quality, direct perceptual access to the world. This means that perception (and by extension, the environment) can be a useful resource, rather than a problem to be overcome by cognitive enrichment. Embodied cognition (in any form) is about acknowledging the role perception, action, and the environment can now play.

A radical conclusion emerges from taking all this seriously: if perception-action couplings and resources distributed over brain, body, and environment are substantial participants in cognition, then the need for the specific objects and processes of standard cognitive psychology (concepts, internally represented competence, and knowledge) goes away, to be replaced by very different objects and processes (most commonly perception-action couplings forming non-linear dynamical systems, e.g., van Gelder, 1995). This, in a nutshell, is the version of embodiment that Shapiro (2011) refers to as the *replacement hypothesis* and our argument here is that *this hypothesis is inevitable once you allow the body and environment into the cognitive mix*. If such replacement is viable, then any research that keeps the standard assumptions of cognitive psychology and simply allows a state of the body to tweak cognition misses the point. To earn the name, embodied cognition research must, we argue, look very different from this standard approach.

## Embodied Cognition: Four Key Questions

The core question in psychology is *why does a given behavior have the form that it does?* The standard cognitive psychology explanation for the form of behavior is that it reflects the contents and operation of an internal algorithm (implemented as a mental representation) designed to produce that behavior on demand (e.g., Fodor, 1975, 2008). The work discussed below replaces complex internal control structures with carefully built bodies perceptually coupled to specific environments. (Of course, embodied cognition solutions will also sometimes require internal control structures. Critically, though, these internal control structures are taking part in the activity of distributed perceptually coupled systems from which behavior emerges online, in real time, in a context. Thus, explicit representations of behavior or knowledge have no place in embodied solutions.)

To get a rigorous handle on this claim, we suggest that there are four key questions any embodied cognition research program must address:

1. What is the task to be solved? Embodied cognition solutions solve specific tasks, not general problems, so identifying how an organism produces a given behavior means accurately identifying the task it is trying to solve at the time. Taking things one task at a time opens up the possibility of *smart* solutions (Runeson, 1977). Organisms using smart solutions solve particular problems using heuristics made possible by stable features of the task at hand, rather than general purpose rote devices which apply algorithms to solve the task. For common tasks, smart solutions are typically more efficient, more stable, and more economical than rote solutions (e.g., Zhu and Bingham, 2008, 2010).
2. What are the resources that the organism has access to in order to solve the task? Embodied cognition implies that there are resources, plural, available to the organism. These resources include the brain but also the body, the environment, and the relations between these things (e.g., the motion of our bodies through the environment). A task analysis should include an exhaustive list of resources available that might contribute, beginning with those available via perception and action and only hypothesizing more complex cognitive resources once the capabilities of these other resources have been exhausted. An exhaustive list is possible if you are able to characterize your task formally; tasks are differentiated from each other in terms of their underlying dynamics (e.g., Bingham, 1995) and thus it is becoming common practice to formalize the task description using the tools of dynamical systems (e.g., Fajen and Warren, 2003; Bingham, 2004a,b; Schöner and Thelen, 2006).
3. How can these resources be assembled so as to solve the task? Solving a specific task means creating a smart, task-specific device that can do the job (Bingham, 1988). To be more specific, it means assembling the required resources into a dynamical system that solves the task at hand as its behavior unfolds over time. Remember, these resources can be distributed over brain, body, and environment. Since we only have access to information about our bodies and the environment via perception, an embodied analysis must include a detailed account of the perceptual information used to connect the various resources (Golonka and Wilson, 2012).
4. Does the organism, in fact, assemble, and use these resources? It is always an empirical question whether the dynamical system hypothesized in step 3 is, in fact, an accurate description of the system the organism has assembled to solve the task. The basic experimental tool for establishing the identity of a dynamical system is the perturbation experiment; systems respond to perturbations of resources in a manner that is specific to the role that resource plays in the system, and this allows you to map the composition and organization of the system at hand (e.g., Kay et al., 1987, 1991; Wilson and Bingham, 2008).

The next sections will review what this new research looks like in practice; we will begin with some simpler cases that tackle and clarify some of our key questions, and end up with two cases of human behavior that demonstrate how to tie these four questions into a coherent research program.

## Embodiment in Action

### Embodiment in action I: Robots

One of the most productive areas to demonstrate the strength of the replacement hypothesis is *robotics*. Robots built on the principles of embodiment are capable of interestingly complex behavior, demonstrating how far you can get without representational enrichment. When you build something yourself from scratch, you know exactly what is (and is not) included in the control systems. This means that your pool of potential explanations for a given behavior is constrained and enumerated, and you can answer questions 2 and 3 in great detail.

#### “Swiss” robots

An early example of embodied cognition robotics comes from Maris and te Boekhorst (1996), who built small Didabots with infra-red detectors placed around their body and a very simple internal control structure: a single rule, “turn away from a detected obstacle.” In this paper, the detector at the front of the robot was deactivated – the robot could no longer “see” anything directly ahead, but it could “see” off to the sides and behind. If it hit an obstacle (a white block) head on, it simply kept moving and pushed the block along until it turned to avoid the next obstacle (either another block or a wall). The first block was then left behind, and the net result (if there was more than one robot at work) was that the randomly scattered blocks were “tidied up” into heaps. This tidying behavior is not specified in the control structure of the robots; it emerges, in real time, from the relationship between the rule, the environment (the size and number of obstacles, the presence, or absence of other robots), and the bodies of the robots (the working front sensors have to be far enough apart to allow a block to fit, or else the robot simply successfully avoids the blocks). Importantly, then, the robots are not actually tidying – they are only trying to avoid obstacles, and their errors, in a specific extended, embodied context, leads to a certain stable outcome that looks like tidying (see also Pfeifer and Scheier, 1999; Pfeifer and Bongard, 2007 for extended reviews of this style of robotics). Understanding the resources the robots had available and how they were organized was what enabled the researchers to identify that the robots were not, in fact, trying to tidy anything up.

#### Locomotion and passive dynamics

Why does walking have the form that it does? One explanation is that we have internal algorithms which control the timing and magnitude of our strides. Another explanation is that the form of walking depends on how we are built and the relationship between that design and the environments we move through.

Considering the resources available to solve this task highlights the centrality of an organism’s design. Humans don’t walk like lions because our bodies aren’t designed like lions’ bodies. The properties of our design are referred to as *passive dynamics* (McGeer, 1990). How are the segments arranged? How are they connected to each other? How springy are the connections? Robotics work on walking show that you can get very far in explaining why walking has a particular form just by considering the passive dynamics. For example, robots with no motors or onboard control algorithms can reproduce human gait patterns and levels of efficiency simply by being assembled correctly (e.g., Collins et al., 2005)^1^. Work at MIT has added simple control algorithms to this kind of system, which allows the robots to maintain posture and control propulsion more independently. The same algorithm can produce a wide variety of locomotion behaviors, depending on which robotic body they control (e.g., Raibert, 1986)^2^. None of these systems include a representation of the final form of their locomotion; this form emerges in real time from the interaction of the passive dynamics with the environment during the act of moving. These robots demonstrate how organisms might use distributed task resources to replace complex internal control structures.

#### Robot crickets

A fascinating example of embodiment in nature has been replicated in the lab in the form of a robot (see Barrett, 2011 for the more detailed analysis of this case that we draw from here). Female crickets need to find male crickets to breed with. Females prefer to breed with males who produce the loudest songs. This means that the task facing female crickets is to find the males who sing the loudest. What resources do they use to solve this task? Female crickets have a pair of eardrums, one on each front leg, which are connected to each other via a tube. Sounds entering from the side activate that side’s eardrum directly, and also travel through the tube from the other eardrum as well. These signals are out of phase if the sound is off to one side, and this increases the amplitude of that side’s eardrum’s response; this arrangement is therefore directional. This explains how the female can tell what direction a sound is coming from, but it doesn’t explain how she uses this information to move toward this sound or how she manages to tune in to crickets of her own species. It so happens that the eardrums connect to a small number of interneurons that control turning; female crickets always turn in the direction specified by the more active interneuron. Within a species of cricket, these interneurons have a typical activation decay rate. This means that their pattern of activation is maximized by sounds with a particular frequency. Male cricket songs are tuned to this frequency, and the net result is that, with no explicit computation or comparison required, the female cricket can orient toward the male of her own species producing the loudest song. The analysis of task resources indicates that the cricket solves the problem by having a particular body (eardrum configuration and interneuron connections) and by living in a particular environment (where male crickets have songs of particular frequencies).

Webb (1995, 1996) have built robots that only have these basic capacities, and these robots successfully reproduce the form of the female cricket’s exploratory behavior. The robots have no stored information about the male cricket’s songs, and simply perceive and act using a particularly arranged body. It is clear that the robot doesn’t explicitly implement “choosing the male with the strongest song”; finding him is simply the result of this embodied strategy operating in the context of multiple male crickets singing and is driven (this robotics work predicts) by the onset of chirps within the song. The success of this work results from carefully analyzing the task at hand, identifying available resources, and specifying how these resources are assembled by the agent (questions 1–3 outlined above).

#### Summary

This robotics work and more like it (e.g., Brooks, 1999; Pfeifer and Scheier, 1999; Beer, 2003; Pfeifer and Bongard, 2007) reveal a great deal of complex behavior (from tidying, to locomotion, to mate selection) can emerge from placing the right type of body into a specific environmental context, without any explicit representation of the form of that behavior anywhere in the system. This work is a proof of concept that embodiment and embedding can therefore *replace* internal algorithms and lead to stable, functional behavior.

### Embodiment in action II: Animals

The robot work is fascinating is one part of a strong argument in favor of the replacement hypothesis. Of course, the next critical step is to establish whether biological organisms actually take advantage of these embodied solutions (question 4) or whether they follow a different, more computational path.

#### Crickets again

Webb’s robot crickets implement a simple embodied perception-action strategy to perform mate selection. A hypothesis that follows from this work is that females use the onset of a male’s song to drive exploration, rather than attending to the entire song and “choosing” the best one. Observation of real crickets shows that female crickets do indeed move before they could possibly have processed an entire song, supporting this embodied “chirp onset” hypothesis (Hedwig and Webb, 2005; see also Barrett, 2011 for an overview).

#### Swarming, herding, hunting

Many animals produce carefully coordinated activities with large numbers of conspecifics. Forming large groups (swarms, or herds, or flocks) is a valuable defense against predators, and maintaining these groups requires ongoing coordination across many individuals. This coordination is not centrally controlled, however, and is not the result of an explicit attempt to maintain a swarm. Instead, the coordination emerges from and is maintained by the operation of straight-forward perception-action coupling rules in a suitable context. Bird flocking is elegantly explained as a coupling between individuals constrained by three principles (Reynolds, 1987): *separation* (avoid crowding neighbors), *alignment* (steer toward average heading of neighbors), and *cohesion* (steer toward average position of neighbors). Interestingly, cohesion exhibits asymmetries that relate to the perceptual capabilities of birds; the average position is a center of mass of only the nearest 5–10 birds, weighted in favor of birds off to the side (reflecting the field of view for bird vision; Ballerini et al., 2007). Sheep herding is similarly straight-forward. Sheep head for the geometric center of the flock when a predator approaches, implementing a “selfish herd” strategy without any individual in the herd being “selfish” *per se* (Hamilton, 1971; King et al., 2012).

A more complex example of coordinated social activity is the pack hunting of wolves. The pattern of their activity, however, is readily explained by two simple rules: (1) move toward the prey until a minimum safe distance is reached, and then (2) move away from any other wolves that are also close to the prey (Muro et al., 2011). No leader is required, no instructions need be given; the form of the group’s hunting activity emerges from a simple perception-action coupling strategy implemented by each individual, operating in a specific context.

Continuing the hunting theme, Barrett (2011) has an extended discussion on what she refers to as “the implausible nature of *Portia*,” the jumping spider. *Portia* is capable of some remarkable feats: deceptive mimicry, creating diversions to distract prey, and taking extended detours in order to sneak up on dinner. This last is especially impressive – detours mean *Portia* must operate for extended periods without direct perceptual contact with its prey animal. This would seem to require some form of route planning (Heil, 1936; Barrett, 2011). As Barrett notes, this hypothesis seemed initially plausible because of the way in which *Portia* scans its environment – prior to taking the detour, it will sit and sway from side to side, seemingly evaluating potential routes and making a selection. However, this scanning behavior, coupled with the anatomy of the spider’s eyes, is actually an embodied strategy that enables *Portia* to generate successful detours using currently available perceptual information (e.g., Tarsitano and Jackson, 1997; Tarsitano and Andrew, 1999); *Portia* is perceiving, not planning.

#### Summary

The advantage of examples from the animal literature is that researchers are less likely to want to attribute performance to complex internal representations (only less likely, of course; the temptation is always there – Kennedy, 1992; Barrett, 2011). However, once we identify that embodied, situated perception-action couplings can produce complex adaptive behavior in other animals, it becomes more difficult to deny the existence of such solutions in our own repertoire unless one wishes to deny the evolutionary continuity between ourselves and the rest of the animal kingdom.

### Embodiment in action III: People

We will now review in some detail two excellent examples of successful replacement style embodied cognition in psychology. These examples are the *outfielder problem* and the *A-not-B error* (see Clark, 1999; Smith and Gasser, 2005 for other uses of these examples). They are useful because (a) they address all four key questions of good embodiment research and (b) both examples have standard cognitive psychology explanations that have been successfully replaced after numerous studies implementing the kind of embodied approach we are advocating for here. These sections will begin by describing the standard cognitive psychology explanations for the outfielder problem and the A-not-B error. We will then take a step back and analyze each task from an embodied cognition perspective, asking our four key questions:

1. What is the task to be solved?
2. What are the resources that the organism has access to in order to solve the task?
3. How can these resources be assembled so as to solve the task?
4. Does the organism, in fact, assemble, and use these resources?

### Embodiment in action III.I: The outfielder problem

How does a baseball outfielder catch a fly ball? There are many factors that make this task difficult; the fielder is far away from the batter, the ball is optically very small and remains so until it is very close to the fielder, the fielder has to move from their starting location to the location where the ball will land at some point in the future, and they have to arrive at this location in time to intercept the ball.

#### The standard explanation

The initial hypothesis is that we catch fly balls by predicting their future location based on the physics of the ball’s motion. A fly ball is an instance of projectile motion, and the physics of this kind of ballistic flight are relatively straight-forward. For an object of a given size and mass, the primary variables that determine the flight are initial direction, velocity, and angle (plus some local constants such as drag, air density, and gravity). Saxberg (1987a,b) suggested that outfielders perceive these initial parameters and then use them as input to an internal simulation (representation) of projectile motion. This representation allows outfielders to *predict* the future location of the ball (Trajectory Prediction). Once the future location of the ball has been predicted, the fielder can simply run to that location and wait.

#### The embodied solution

Saxberg’s (1987a,b) solution assumes that the act of catching a fly ball is a lot like solving a physics problem, relying on some limited resources (the ball’s initial conditions) and some internal simulation. In contrast, the embodied solution first asks if that’s true by asking “What *are* the resources that are available in this task, and how might they help a person trying to catch a ball?”

#### What is the task to be solved?

A fielder stands in the outfield of a baseball diamond, around 250 ft from home plate. The batter pops a fly ball (projectile motion along a parabolic trajectory) into the air and the fielder must locomote from where they are, to where the ball will be when it hits the ground (hopefully in time to catch it before it hits the ground). So, the fielder’s task is to move themselves so that they arrive at the *right place* at the *right time* to intercept a *fly ball*. Sometimes fielders are in a direct line with the flight of the ball, but the general problem to be solved involves the fielder being *off to one side*.

#### What are the resources available?

The first thing to note is that, at the distances involved, the optical projection of the baseball is tiny. Any attempt to figure out how far away the ball is and where it’s going using changes in optical projection size will be riddled with errors (if it’s possible at all; Cutting and Vishton, 1995). These errors would propagate through any simulation, which makes solutions based on computing simulations of projectile motion unstable. This means that the simulation solution is not a likely resource (and in fact the evidence suggests it is not an option; Shaffer and McBeath, 2005). What else is available?

To identify the full range of available resources, we need to understand the physical properties of the fly ball event. Events unfold over time, and are distinguished from one another by their underlying *dynamics* (which describe both how the system changes over time and the forces which produced the change; Bingham, 1995). In the present example, the relevant dynamics are that of projectile motion. As a given example of the projectile motion dynamic plays out, it creates *kinematic* information which can be detected and used by an observer. Kinematic descriptions include only how the system changes over time, without reference to the underlying forces. Perceptual systems can only detect kinematic patterns, but observers actually want to know about the underlying dynamic event; this is the perceptual bottleneck (Bingham, 1988). Kinematics can *specify* the underlying dynamics, however (Runeson and Frykholm, 1983) and detecting a specifying kinematic pattern is equivalent to perceiving the underlying dynamic (solving the bottleneck problem and allowing direct perception as suggested by Gibson, 1966, 1979). The information that an outfielder might use to continuously guide their actions to the future position of the ball must therefore be kinematic and specific to this future position.

The batter provides the initial conditions of the ball’s trajectory (direction, velocity, and angle) and, after that, the flight unfolds according to the dynamics of projectile motion. This dynamic produces motion along a parabolic trajectory. The form of this motion is that the ball initially rises and decelerates until it reaches a peak height when its velocity reaches zero; it then accelerates as it falls down the other side of the parabola. This motion is the kinematic information that is available to the observer.

The fielder also brings resources with them: these include the ability to detect kinematic information and (most usefully) to locomote over a range of speeds along any trajectory across the field.

#### How might these resources be assembled to solve the task?

How can the perceptual information specifying the dynamics of the fly ball be used in conjunction with the fielder’s ability to perceive kinematics and locomote? The parabolic flight of the ball creates the possibility of two basic solutions. Each strategy requires the outfielder to move in a particular way so as to offset some aspect of the parabolic flight, either the acceleration or the curve of the path. If the fielder is able to successfully offset either the acceleration or the curve of the path, then they will end up in the right place in the right time to intercept the ball. When reading about these solutions in more detail below notice that neither one requires the fielder to predict anything about the ball’s future location, only to move in a particular way with respect to the ball’s current motion; this is *prospective control* (e.g., Montagne et al., 1999).

The first solution is called optical acceleration cancelation (OAC; e.g., Chapman, 1968; Fink et al., 2009) and requires the fielder to align themselves with the path of the ball and run so as to make the ball appear to move with constant velocity. The second strategy is called linear optical trajectory (LOT; e.g., McBeath et al., 1995) and requires the fielder to move laterally so as to make the ball appear to trace a straight line. Which strategy is adopted depends on where the fielder is relative to the ball (OAC works best if the ball is coming straight for you, LOT allows you to intercept a ball that is heading off to one side).

#### Does the organism, in fact, assemble, and use these resources?

The computational strategy suggests that the outfielder will run in a straight line to the predicted landing site. This is because the fielder computes the future landing site based on input variables that the fielder detects before setting off. Since the shortest path to a known landing site in open terrain is a straight line, the fielder should run directly to the place where they intercept the ball. Outfielders do not typically run in straight lines, ruling the computational strategy out. LOT and OAC predict either a curving path or one with a velocity profile that offsets the acceleration of the ball. The evidence generally favors LOT (e.g., McBeath et al., 1995) but there is evidence that OAC is a viable and utilized strategy under certain conditions (e.g., Fink et al., 2009).

These solutions have numerous advantages over the computational solution. First, instead of relying on an initial estimate of the ball’s motion, which could be in error, they allow the fielder to continuously couple themselves to the ball. This coupling provides fielders with numerous opportunities for error detection and correction. Second, the strategies provide a continuous stream of information about how well the fielder is doing. If the ball still seems to be accelerating, or if its trajectory is still curved, this tells the fielder both that there is an error and what to do to fix the error. If the fielder is running flat out and is still unable to correct the errors, this specifies an uncatchable ball, and the fielder should switch to intercepting the ball on the bounce instead. The affordance property “catchableness” is therefore continuously and directly specified by the visual information, with no internal simulation or prediction required.

#### Summary

In both LOT and OAC, various task resources (the motion of the ball, the fielder, and the relation between them specified by the kinematics of the ball viewed by the moving observer) have been assembled into a *task-specific device* (Bingham, 1988) to solve the task at hand (intercepting the projectile). This assembly is *smart*, in the sense described by Runeson (1977); it takes advantage of certain local facts of the matter to create a robust but task-specific solution (neither LOT nor OAC are a general solution to the problem of interception, for example). The most important lesson here is that the relation between perceptual information (about the motion of the ball) and an organism (the outfielder) *replaces* the need for internal simulation of the physics of projectile motion.

### Embodiment in action III.II: The A-not-B error

What do children know about objects and their properties, and when do they come to this knowledge? Piaget (1954) investigated this question by asking children of various ages to search for objects that were hidden behind some obstacle in view of the children. Prior to about 7 months, children simply don’t go looking for the object, as if it has ceased to exist. From around 12 months, children will happily go and retrieve the hidden object, seemingly now understanding that even though they can’t see the toy they want, it’s still there to be found. In the transition, however, children make a rather unusual “error” – after successfully reaching several times for a hidden object at a first location A, they will then fail to reach for the object hidden at location B, even though the hiding happened in full view of the object. They will instead reach to A again (hence “A-not-B error”).

There are a variety of standard cognitive explanations for this error, but all in essence assume that (a) the child has developed the necessary object concept that includes the knowledge that objects persist even when out of view but (b) there is something about reaching that cannot tap that knowledge reliably. The child’s underlying *competence* can be demonstrated using looking behavior as a measure, for example; children look longer at displays showing the error trial, suggesting they know something is not right (e.g., Baillargeon and Graber, 1988). The problem, therefore, is in the reaching *performance*: reaching cannot yet access the knowledge necessary. This performance-competence distinction is a common theme in the cognitive developmental literature. It assumes that the goal of the science is to understand the core competence, and that to do so you must devise clever methods to bypass the potential limitations of performance.

Thelen et al. (2001) challenged every single aspect of this account with their embodied dynamical systems model of the reaching task. This model was the end result of numerous experiments motivated by a rejection of the performance-competence distinction and a renewed focus on the details of the task at hand. As Thelen et al. put it, “The A-not-B error is not about what infants *have* and *don’t* *have* as enduring concepts, traits, or deficits, but what they *are* *doing* and *have* *done*” (p. 4). The end result was an account of the A-not-B error that replaces object knowledge and performance deficits with the dynamics of perceiving and acting over time in the context of the reaching task.

#### What is the task to be solved?

This is actually quite a complicated question. The canonical version of the task requires the infant to watch as an attractive toy is hidden at location A. The child is then allowed to search for and retrieve the object several times, after which the object is hidden at location B in full sight of the baby.

One of the inspirations for pursuing a dynamical system, embodied approach here was that almost every parameter of this task is known to affect infants’ performance. These parameters include the distance to the targets, the distinctiveness of the covers, the delay between hiding and search, what the infant is searching for (food or a toy), whether the infant is moved and how much crawling experience they have (see Thelen et al., 2001 for a detailed overview). If the A-not-B error reflects object knowledge, why do these factors matter so much?

To get a handle on this question, the first thing that Thelen et al. (2001) did was to enumerate the details of the canonical task (Section 2.2) so that they had a clear understanding of the available resources that might impact infants’ performance. First, the infant gets *continuous visual input* (Section 2.2.1) from two wells in a box placed a certain distance away from the child and apart from one another. The experimenter draws the infant’s attention to the object, and then hides the object in well A. This *specific visual input* (Section 2.2.2) indicates which well the reaching target is in. After a short *delay* (Section 2.2.3) during which infants typically look at the cued location, they perform a *visually guided* *reach* (Section 2.2.4) to retrieve the object. This reach requires them to *remember* (Section 2.2.5) the location of the hidden object for the duration of the delay. This is repeated several times until the switch to the B location, at which point the infants make the error around 70–80% of the time (depending on their *developmental status*; Section 2.2.6).

#### What are the resources available?

In this version of the task, the resources that might impact performance include the details of the continuous and specific visual input, the length of the delay, and the delay’s relationship to the temporal dynamics of the memory of the previous reaches. The infant also brings resources to the task. For instance, their performance depends on their ability to maintain visual attention and the way in which they perform visually guided reaches. Thelen et al. (2001) do not include an object concept as a resource. The purpose of this seeming omission is to see how well they can model the behavior without invoking any core competence separate from observed performance.

#### How might these resources be assembled to solve the task?

The reason why this work by Thelen et al. (2001) is such a powerful example of replacement style embodied cognition is that their model is an excellent example of using dynamical systems to explain how perceptual and embodied resources might be assembled to produce an error that, on the face of it, seems to require a representational explanation (in the form of an infant’s object concept). The model specifies two locations in a metric field representing the infant’s reach space and takes specific perceptual input about where to reach. This input raises activation at the appropriate location in the motor planning field and generates a reach in the right direction once a threshold is crossed. Reach direction planning unfolds continuously over time using population coding (c.f. Georgopoulos, 1995). Activation in this field has a temporal dynamic that prevents it from fading immediately; the movement planning field has memory about its recent behavior. Activations at different locations in the field interact, allowing for competition and cooperation between them. The model is initialized and presented with specific input; the behavior of the model emerges as the various competing dynamics (specific input, task input, memory, reach planning, etc.) unfold and change the shape of the field controlling reaching. By the time the specific input is switched to location B, the field has taken on a shape which reflects this competition, and the perceptual input from B is effectively being detected by a very different system than the one which first detected input from location A. Its behavior is correspondingly different; specifically, if the parameters match the canonical version of the task, the model will make the A-not-B error. Note there is no mention of an “object concept” in the model specification. Yet, the model is able to re-create the A-not-B error simply by implementing a reach system with its own dynamical properties.

#### Does the organism, in fact, assemble, and use these resources?

The model is extremely successful at capturing the key phenomena of the A-not-B task. It also captures how performance is affected by changes to task details (e.g., variation in reach delay, changes in object properties). Object concept based explanations have been proposed for these effects (e.g., see Diamond’s, 2001 response to Thelen et al.’s, 2001 target article). However, there are other aspects of task performance that object concept explanations struggle to cope with. Most interestingly, the model predicts and then explains the novel experimental finding that the A-not-B error occurs *in the absence of hidden objects* (Smith et al., 1999). If there is no object to remember, then object concept based explanations are at a loss to explain why the error persists; after all, there is no object to conceptualize. In contrast, the embodied model predicts that the “error” comes from the immature dynamics of reaching, and not an incomplete object concept. This then suggests that you should be able to generate the error in older children by increasing the complexity of the reaching requirements. Consistent with this, Smith et al. (1999) and Spencer et al. (2001) generated the error in 2 year olds and similar reach biases have been observed in children up to 11 (Hund and Spencer, 2003) and even adults (Spencer and Hund, 2002). There is no clear reason to expect these biases on the basis of an object concept explanation. The best explanation for this pattern of results is that the observed reaching behavior does indeed emerge from the kind of embodied task dynamic described by the model.

#### Summary

The A-not-B task has a long history of explanations based in standard, representational cognitive psychology. These explanations assume that the reach is an error caused by an incomplete object concept, to which the immature motor system has limited access until around the age of 12 months. Thelen et al.’s (2001) embodied approach *replaces* the object concept with the dynamics of reaching to grasp and successfully accounts for the wide variety of context effects, as well as explaining novel versions of the error generated without any hidden objects and in older children.

### The conceptualization hypothesis for embodiment: Concepts and grounding

We have identified embodied cognition as a cluster of research tied together by the same basic research strategy; (1) identify the task at hand, (2) identify the resources available within that task space that might help an organism solve the task, (3) generate hypotheses about how these resources are assembled and coordinated (perhaps formalizing this hypothesis in a model; see Bingham, 2001, 2004a,b for another example, and Golonka and Wilson, 2012 for a detailed analysis of that model), and finally (4) empirically test whether people, indeed, use these resources assembled in this way. This is not, however, the only style of research going under the banner of embodiment, and it’s fair to ask on what basis we are ruling this other research out from our classification.

Many examples fall under what Shapiro (2011) calls the *conceptualization hypothesis*. This is the hypothesis that how we conceive of our world is grounded in and constrained by the nature of the perception-action systems that we are (our bodies). For example, Lakoff and Johnson (1980, 1999) describe how common metaphors are typically grounded in the nature of our bodies and experiences in the world (the future is *forward*, power is *up*, relationships are *a journey*). This style of research doesn’t seek to replace the concept with a different process. Instead, it looks to find examples where use of the concept can be primed or altered by manipulations of the grounding state of the body.

There are many recent examples of this type of research in the literature; we will briefly focus on two representative studies. The first claims to demonstrate how a state of the body affects our access to a mental representation for magnitude estimation (Eerland et al., 2011) while the second claims to show an effect in the other direction, with a mental state biasing the body state the mental state is supposedly grounded in Miles et al. (2010).

#### Leaning to the left makes the Eiffel Tower seem smaller

People can generate sensible estimates of the magnitude of things, such as the height of the Eiffel Tower, even when they don’t know the exact answer. These magnitudes are hypothesized to be generated by a mental representation of magnitudes organized like a number line, with small numbers at the left end and larger numbers to the right (Restle, 1970). Eerland et al. (2011) had people stand balanced slightly to the left or to the right of center to test the hypothesis that this postural bias would make either the left or right end of the number line more accessible. If it did, then people should be primed to generate lower estimates of magnitude when leaning left and greater ones when leaning right.

The results were mixed. When people leaned left they did, on average, make slightly smaller estimates than when leaning right and the authors concluded that these data support the hypothesis; access to the mental number line, arranged left to right, is, at least, partly grounded in the left to right sway of the body. It should be noted, however, that the effect size was very small, the effect was not observed for all the questions, and there was no effect of leaning to the right.

#### Thinking about the future makes you sway forward

The second example of conceptualization style research is Miles et al. (2010), who had people engage in “mental time travel” by thinking about events in either the past of the future. They measured postural sway at the knee, and found that as people thought about the future this sway was biased toward the front (the future is *in front*). When people thought about events in the past, their sway was biased backward (the past is *behind*). Again the effect was small (peaking at a bias of approximately 2 mm in each direction) but the authors concluded that their data demonstrate a connection between the state of the body and the contents of the cognitive representation of time.

#### Where is the embodiment?

Neither of these studies begins with a task analysis and neither considers what perceptual and embodied resources are available to solve the task. This eliminates the opportunity to discover what substantive role these resources can play in cognition. Instead, the assumption made in both these studies is that the task is solved internally, representationally, by a cognitive process that can tweak or be tweaked by a state of the body. There isn’t any compulsory, critical, constitutive role for the body and environment in the proposed mechanism for solving the task at hand, as there is in all the other work reviewed. You cannot catch a fly ball without moving. The fielder’s movement *inevitably* creates the information for either LOT or OAC, which can then structure the observed behavior. You cannot do the A-not-B task without reaching. Reaching *inevitably* invokes the dynamics of visually guided reaching, which can then structure the observed behavior. You can, however, lean left and not have it affect your estimates of magnitude, and you can think about the future without leaning forward. Conceptualization style embodiment research does not identify the body as a task-critical resource, nor does it generate any formal account of how the body forms part of a task-specific solution to the task at hand. At best, it demonstrates that sometimes thoughts and actions go together.

## Taking the Next Step – An Embodied Analysis of Language

This paper has laid out what we propose is a necessary research strategy for a genuine embodied cognitive science. We’ve looked at a progression of existing research that follows this strategy, beginning with simple robotic systems up through non-human animal behavior, and on to two cases of human behavior – one straight-forward perception-action system (catching a fly ball) and one more traditional cognitive task (the A-not-B task). The point was to show that this approach is productive across a wide variety of tasks and behaviors, and that it demonstrates the kind of continuity evolutionary theory tells us exists across biology.

We would like to round this article out with an initial foray into an embodied analysis of that classic cognitive task, *language*. Our goal here is simply to take what we think is the first step: identifying the nature of a critical resource present in a language event, specifically the form and content of *linguistic information*. This can then guide and constrain the non-representational empirical investigations that we hope will follow.

### Language: It’s special, but it’s not magical

Most psychologists generally assume that *catching* a fly ball and *talking about* catching a fly ball are two different *kinds* of task, in the sense that you can’t use the tools appropriate to studying how to catch a fly ball to understand how we communicate through language. Language is a very interesting kind of behavior, and it has some properties that make it very special. But it is not magical; it is a product of evolution, the same as the rest of our behavior, so it makes perfect sense to expect it to be amenable to the analyses that have been so successful in other domains. In other words, our first move is simply to treat perception-action problems and language problems as the same kind of thing.

As we will discuss shortly, there is one important difference to worry about, specifically in how perceptual and linguistic information come to have their meaning. This difference, however, can only be seen by the third person, scientific analysis of the situation. An embodied approach should never forget that it’s trying to explain the first person experience of the organism (a point made forcefully by Barrett, 2011) and from this perspective *there is no difference at all* between the two types of information. In its day-to-day life the organism never gets to “peer behind the curtain” – kinematic patterns in energy arrays are all we ever have access to. The job of the learning organism is to detect these patterns, and come to learn what they mean by using that information to do something. If you can use some information to intercept a fly ball, then you have demonstrated that you know that that’s what the information means. Similarly, if you can use linguistic information to reply correctly to an interlocutor, you have again demonstrated that you know that that’s what the information means. The basic process is the same; learn to detect the relevant structure and learn to use it appropriately.

### How information gets its meaning

Events in the world are identified by their underlying dynamics; these dynamics create kinematic patterns in energy arrays and these patterns can serve as *perceptual information* about the dynamics that created them (Bingham, 1995). For perception, structure in an energy array is *about* the dynamic event in the world that created the structure in the moment (for example, the optical information created by the motion of a fly ball is about the motion of the fly ball). This relationship is underwritten by ecological laws (Turvey et al., 1981) and detecting the information allows the organism to perceive the dynamical event.

Every language event (speech, writing, gesture) also creates structure in energy arrays (speech creates acoustic structure; writing and gesture creates optical structure). To an organism capable of language use, this structure can serve as *linguistic information*, and because we are treating them as the same kind of thing, we can analyze linguistic information the same way we analyze perceptual information. The only difference between perceptual information and linguistic information is in the relationship between the structure in the energy array and the meaning of the information. For language, the structure in the energy array is not about the dynamics of, say, articulation; it’s about whatever the words mean. The structure comes to have this meaning because of the social conventions of the language environment and what we learn is, therefore, a *conventional* meaning of the pattern. This conventional underpinning gives stability to linguistic information, but the difference between a law and a convention is very important. Conventions can change and so can the meaning of words; language is much less stable than perception. This decreased stability is, of course, a fact of language to be explained, so perhaps it is not a disaster for the analogy we are developing here.

### Do we need representation?

This is the point where standard cognitive science usually jumps in and claims that conventional meaning requires representational support. Linguistic information is created by the unfolding of a complex dynamic in the present time, but the meaning of this information is the conventional one that may be about something not present at that time; we can talk about things in their absence in a way that has no analogy in perception. So in what sense can linguistic information have meaning if not in the form of internal models of the people, objects, places, etc., to which the words refer?

This sticking point is, to some extent, a product of the form of the question. To ask what a word means implies something static and internal – words *have* meanings. So, our approach here is to ask the same question in a different way. As we said earlier, if someone is able to respond appropriately to linguistic information, then it is fair to say that this person knows what the information means. Instead of asking how we learn the meaning of words, we can ask, instead, how do we learn to use and respond to linguistic information? Can we respond appropriately to linguistic information without possessing mental representations? As discussed in the previous sections on robotics, quite interesting, and complex behavior can emerge without explicit internal models of it. Still, none of these robots used language.

In perception, the argument goes, representations are not necessary because the specification relationship between perceptual information and the world makes perceiving the information identical to perceiving the world (Gibson, 1966, 1979; Turvey et al., 1981). What this means is that organisms can respond appropriately to perceptual information without the need to cognitively enrich the perceptual input. The critical issue for language is whether the conventional relationship between linguistic information and what that information is about is sufficient to support something like direct perception.

Chemero (2009) has an extensive argument about how convention can indeed be sufficient, as part of his suggestion that even perceptual information can be grounded in convention. Specifically, he uses conventions as defined by the situation semantics of Barwise and Perry (1983) and we suggest that this analysis will be the place to begin to address this question in the future. To summarize the key points, Barwise and Perry proposed that information is created for organisms by *situations*; a given situation will be an instance (token) of a type of situation, and situations can be connected by constraints. If two types of situation, S1 and S2, are connected via a constraint, then a token S2 is informative about a token S1 by virtue of that constraint. An organism has access to that information if and only if they have access to one of the tokens and the constraint. This is precisely the case in the example of language. If S1 is “the situation being discussed” and S2 is “the language event of the discussion,” these are connected by the constraints of the local language environment. By this account, a token of S2 (e.g., the utterance “the rain in Spain stays mainly in the plain”) is informative about a token of S1 (the typical pattern of rain fall in Spain) but only to a skilled user of the English language. If the utterance was instead “La lluvia en España se mantiene principalmente en la llanura,” our English language user would not be informed about S1 because they don’t have access to the relevant constraints of Spanish. Situation semantics provides a formal language for talking about how linguistic information can be informative about the world even despite its basis in convention. There is much work still to do here, but as Chemero (2009) notes, this framework has the benefit of treating specifying and conventional information as the same kind of thing and it therefore seems like a good place to start a non-representational account of language meaning.

It is worth saying outright that arguing against the need for representations to support language is not the same thing as claiming that the brain has no role in language. The brain is clearly involved (as it is involved in perception/action) and an embodied approach to language will need to engage with this fact, so long as hypotheses about what the brain is doing are consistent with the embodied analysis we are applying here. For example, there is a literature on the coupling between articulation and neural dynamics as a mechanism for language comprehension. This work focuses on the production of syllables and models that in terms of oscillator dynamics which can then be coupled to the oscillator dynamics of the cortex (Luo and Poeppel, 2007; Giraud and Poeppel, 2012; Peelle and Davis, 2012). There is some dispute about whether the syllable is the correct phonetic level of analysis (Cummins, 2012), but regardless, the form of this argument matches parts of the analysis we propose here. In particular, this framework suggests a way to link linguistic information to cortical dynamics. Thus, in principle, there is no need to invoke representations to explain how linguistic information can precipitate actions. The non-representational alternative is a non-linear dynamical system where structure in energy arrays (in the form of perceptual and linguistic information) cause changes in cortical dynamics, which are coupled to limbs, mouths, etc., capable of taking action. Taking action (moving, speaking) changes the landscape of perceptual and/or linguistic information, which impacts the cortical dynamics, and so on.

### Language, though special, is amenable to an embodied analysis

We create linguistic information (e.g., speech or written text) to achieve goals (e.g., directing and regulating the behavior of ourselves and others). The dynamical system creating linguistic information entails the coupled dynamics of the articulators and the brain, both of which are nested in a socially defined language environment with its own dynamical properties. Language dynamics are therefore complex and defined across multiple coupled dynamical systems, but linguistic information is still being created by a dynamical event the same way perceptual information is; they are not different in kind.

This information is a critical task resource, in exactly the same way as perceptual information is a critical task resource. In fact, we argue that the similarities between the two are strong enough to import the analyses used with perception directly over to an analysis of language. The most important similarity is that *from the first person perspective of a perceiving, acting language user, learning the meaning of linguistic information, and learning the meaning of perceptual information is the same process*. The differences in the behavior supported by these two types of information (which are, indeed, important) arise from the differences in the way these two types of information come about and connect to their meaning. But the similarities mean the same basic approach to studying how we use information to perceive meaning can apply to language as much as to perception and action; a step forward in and of itself.

Although language is clearly a tremendous step up in terms of the complexity of the dynamics involved, the essential form of the analysis can remain the same. Linguistic information is a task resource in *exactly* the same way as perceptual information is a task resource, and we should treat it as such when we try to figure out how it fits into the task-specific device an organism is forming to solve a given problem. We suggest that it is vital to exhaust this strategy *first*, before leaping to the conclusion that it simply can’t be done without the representations that many other cognitive systems just don’t seem to require.

### Other embodied approaches to language: Another note on grounding

This is not the first attempt to embody language, but the previous efforts are more in line with the conceptualization hypothesis we reviewed above and suffer from the problems we highlighted there (as well as others; see Willems and Francken, 2012). They hypothesize that meaning is grounded in a simulation of previous experiences, a simulation which would include embodied elements of those previous experiences. Tasks measuring comprehension should reflect the presence of this kind of simulation (Barsalou, 1999). Two high profile attempts to measure these embodied simulation effects are the *action-sentence compatibility effect* (e.g., Glenberg and Kaschak, 2002) and the *sentence-picture verification task* (e.g., Stanfield and Zwaan, 2001).

#### Action-sentence compatibility

Glenberg and Kaschak (2002) had participants rate whether sentences were sensible. Some of the sentences implied a directional movement (e.g., “close the drawer” implies a movement away from the person). Participants responded by moving to press a button, and the movement was either compatible or not with the implied direction in the sentence. Participants were faster when the response direction and the implied direction were compatible, and slower when they were not. The authors suggest that this demonstrates people are mentally simulating the action in the sentence in order to comprehend the sentence; “language understanding is grounded in bodily action” (Glenberg and Kaschak, 2002, p. 562).

#### Sentence-verification task

Stanfield and Zwaan (2001) tested the simulation hypothesis by providing people with sentences that implied an orientation for an object, e.g., “the pencil is in the cup” implies a vertical orientation while “the pencil is in the drawer” implies a horizontal orientation. They then showed people a picture of the object in a compatible or incompatible orientation and asked people to verify if the pictured object matched the sentence; participants were faster to respond in the compatible condition and vice versa.

The major problem with this research is that it again assumes all the hard work is done in the head, with perception and action merely tweaking the result. Before this type of research can tell us anything meaningful about language comprehension, more work must be done to answer some basic questions. There is no account of the resources that exist in the task presented to participants, and this is a critical part of identifying what the task is from the participants’ perspective. For example, what is the information content of a picture of an object, what are the dynamics of button pressing behavior (or any response type being used), and what is the relationship between these two things – what happens if you try to control the latter using the former? These are not easy questions; for example, Gibson himself highlighted how difficult it is to establish exactly what the information content of a picture of something actually is (Gibson, 1979). But without this, you cannot begin to explain how hearing different sentences influences a button press response to those pictures. There may indeed be a story there; after all, the results have been demonstrated multiple times. But it is a story remaining to be told, and as in the rest of the work surveyed here, we think that the answer to these questions will likely lead to mental simulations being replaced the relevant dynamics identified by a task analysis.

## Conclusion

At the beginning of the twentieth century, a German teacher named Wilhelm von Osten owned a horse called Hans. Hans, he claimed, could count and do simple maths and he demonstrated this ability for several years in free shows. It wasn’t until psychologist Oskar Pfungst tested this claim rigorously that the truth was revealed: Hans did not know maths, but he did know to stop tapping his hoof when his owner indicated that he had reached the correct answer (by visibly but subconsciously relaxing; von Osten was not a fraud). Abstract knowledge such as how to add is typically seen as requiring some form of internal representational state, but here, the cognitive explanation (that Hans had the internal ability to count) was *replaced* by a straight-forward perceptual coupling to his environment.

The story of Clever Hans has stood as a cautionary tale in psychology ever since; identifying an organism’s actual solution to a problem requires the ability to identify *all* the potential solutions to a task followed by careful experimental testing to identify which of all the possible options are actually being used. This remains as true now as it did in 1907 when Pfungst ran his tests.

Standard cognitive science proceeds under two related assumptions that interfere with its ability to identify the actual solutions. These are poverty of stimulus, and the consequent need for internal, representational enrichment of perception. The objects and processes of standard cognitive psychology have a specific job to do that reflects the hypothesized need to enrich perceptual information. But these assumptions mean that cognitive research never even tests the genuinely embodied alternative solutions we now know are viable options.

Replacement style embodied cognition removes these assumptions and instead looks at all the resources in the environment that might support complex behavior and, critically, the information that might serve to tie them together. One of the most important discoveries of the last 40 years has been that there is, in fact, rich and varied information in the environment (Gibson, 1966, 1979)^3^ that we are able to use to produce all manner of complex behaviors. The availability of this high quality perceptual information removes the need to invoke any additional cognitive constructs to explain interesting behaviors. Our behavior emerges from a pool of potential task resources that include the body, the environment and, yes, the brain. Careful analysis is required to discover exactly which of these resources and the relations between them form the actual solution used to solve a given task.

It is true that replacement style embodied cognition cannot currently explain everything that we do (Shapiro, 2011). Even some of the most enthusiastic researchers in embodied cognition think that there are “representation hungry” problems, which simply cannot be solved without something like an object or process from standard cognitive psychology (Clark and Toribio, 1994); language is the major case here. We are more optimistic. All that we can really conclude at this time is that replacement style embodied cognition cannot explain these problems *yet*. We believe that there is no principled reason why these behaviors cannot be explained with replacement style embodied solutions, given that human beings are, we think, best described as the kind of perceiving, acting, embodied, non-linear dynamical systems doing the replacing. This optimism reflects the successes we’ve described here, and especially the fact that when embodied cognition researchers *have* turned their attention to “representation hungry” problems, they have actually had great success. The embodied analysis of the A-not-B error remains the best example of this; it literally replaces “thinking about things in their absence” with embodied action. Another example is the work with *Portia* spiders (see above and Barrett, 2011 for a review). We have suggested a further step forward here, with an initial analysis of language that replaces what words *mean* with what language lets us *do*; of course, it remains to be seen if this is as successful (but, see also Port and Leary, 2005; Port, 2007; for more on tackling language).

Replacement style embodied cognition research has produced methods, formal tools (primarily in the form of dynamical systems models) and a great number of empirical successes. The explanations it produces place embodiment at the *center* of the organism’s solution to a given task, rather than on the periphery, and this is the research we feel deserves the name embodied cognition.

## Conflict of Interest Statement

The authors declare that the research was conducted in the absence of any commercial or financial relationships that could be construed as a potential conflict of interest.

## Footnotes

^1^http://ruina.tam.cornell.edu/research/ for videos and more details of these robots.

^2^http://www.ai.mit.edu/projects/leglab/ for videos and more details.

^3^Three recent reviews of how Gibson’s work in visual perception underpins much of the embodied cognition literature include Barrett (2011), Chemero (2009), and Shapiro (2011).
